# Shape oscillations of particle-coated bubbles and directional particle expulsion†
†Electronic supplementary information (ESI) available. See DOI: 10.1039/c6sm01603k


**DOI:** 10.1039/c6sm01603k

**Published:** 2016-09-05

**Authors:** Vincent Poulichet, Axel Huerre, Valeria Garbin

**Affiliations:** a Department of Chemical Engineering , Imperial College London , London SW7 2AZ , UK . Email: v.garbin@imperial.ac.uk

## Abstract

Bubbles stabilised by colloidal particles can find applications in advanced materials, catalysis and drug delivery. For applications in controlled release, it is desirable to remove the particles from the interface in a programmable fashion. We have previously shown that ultrasound waves excite volumetric oscillations of particle-coated bubbles, resulting in precisely timed particle expulsion due to interface compression on a ultrafast timescale [Poulichet *et al.*, *Proc. Natl. Acad. Sci. U. S. A.*, 2015, **112**, 5932]. We also observed shape oscillations, which were found to drive directional particle expulsion from the antinodes of the non-spherical deformation. In this paper we investigate the mechanisms leading to directional particle expulsion during shape oscillations of particle-coated bubbles driven by ultrasound at 40 kHz. We perform high-speed visualisation of the interface shape and of the particle distribution during ultrafast deformation at a rate of up to 10^4^ s^–1^. The mode of shape oscillations is found to not depend on the bubble size, in contrast with what has been reported for uncoated bubbles. A decomposition of the non-spherical shape in spatial Fourier modes reveals that the interplay of different modes determines the locations of particle expulsion. The *n*-fold symmetry of the dominant mode does not always lead to desorption from all 2*n* antinodes, but only those where there is favourable alignment with the sub-dominant modes. Desorption from the antinodes of the shape oscillations is due to different, concurrent mechanisms. The radial acceleration of the interface at the antinodes can be up to 10^5^–10^6^ ms^–2^, hence there is a contribution from the inertia of the particles localised at the antinodes. In addition, we found that particles migrate to the antinodes of the shape oscillation, thereby enhancing the contribution from the surface pressure in the monolayer.

## Abstract 2

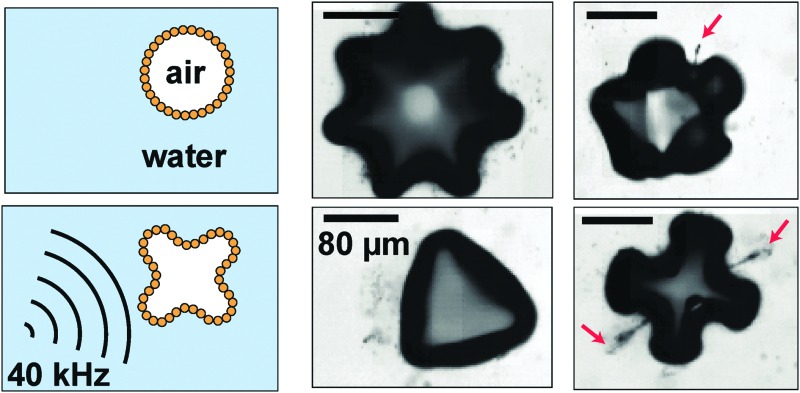
Ultrasound waves drive shape oscillations of particle-coated microbubbles. During the ultrafast, non-uniform deformation of the interface, particles are expelled from the antinodes of the shape oscillations.

## 1 Introduction

Solid particles adsorbing at fluid–fluid interfaces are widely exploited to stabilise emulsions and foams.^1,2^ Because the energy cost of removing a colloidal particle from a fluid–fluid interface can be up to millions of times the thermal energy for micron-sized particles,^3^ adsorption can be considered to be irreversible. The outstanding stability of colloidal particles at fluid interfaces has enabled the development of a range of novel materials, such as colloidosomes,^4^ armoured bubbles,^5^ bijels,^6–8^ bijel particles,^9^ and capillary foams,^10^ which exhibit complex structures and exceptional mechanical properties.^11^ Alongside these emerging applications, recent advancements include fundamental studies of colloidal interactions at interfaces,^12,13^ and of the structure and mechanics of colloid monolayers.^14,15^ Furthermore, newly developed theoretical models^16^ and numerical simulation methods^17^ are providing new insights into the dynamics of particle–laden interfaces.

Particle-stabilised bubbles, or armoured bubbles, present new opportunities in controlled release for medical applications, and in functional materials.^18^ For applications in controlled release, it is desirable that the coating of particles be forced to desorb in a programmable fashion when an external stimulus is applied. Particle desorption can be triggered by addition of surfactants,^19,20^ by changing the pH or electrolyte concentration,^21,22^ by magnetic or gravitational forces,^23,24^ or by interface compression.^25–27^ One of the most common triggers for drug delivery applications is ultrasound.^28^ We have recently shown that ultrasound waves can drive particle-coated bubbles into highly dynamic deformation, triggering particle desorption by interface compression on a ultrafast timescale.^26^ This method holds promise for controlled release since desorption is programmable in time, the payload of particles can be released in under a millisecond, and physicochemical modification of the particles or the fluids is not required.

During ultrasonic driving, bubbles undergo volumetric oscillations, and above a certain threshold in acoustic pressure, shape oscillations can develop.^29^ In our previous work we have shown that particle-coated bubbles also exhibit both these behaviours, and that both scenarios lead to particle expulsion.^26^ When a bubble remains spherical during volumetric oscillations, particle expulsion is primarily due to the decrease in area during the compression phase, which results in a sufficiently large surface pressure within the particle monolayer to overcome the desorption energy.^25^ When a bubble undergoes shape oscillations, we found that desorption is strongly localised at the antinodes of the shape oscillation, that is, the points where the amplitude of the radial excursion is a maximum.^26^ In this case, additional mechanisms can promote desorption. Due to the high frequency of ultrasonic driving (10–100 kHz) the radial velocity, **, and acceleration, *R*, of the interface are very large, of the order of 1 ms^–1^ and 10^5^ ms^–2^ respectively, and can influence particle desorption. In this paper, we investigate the conditions for the occurrence of shape oscillations of particle-coated bubbles, and the mechanisms of directional particle desorption.

## 2 Dynamics of uncoated and coated bubbles in ultrasound

### 2.1 Spherical oscillations

The pressure fluctuations created by an ultrasound wave, *p*(*t*) = *p*_a_ sin(*ωt*), with *p*_a_ the acoustic pressure amplitude and *ω* the angular frequency, cause gas bubbles to periodically compress and expand. For volumetric oscillations of sufficiently small amplitude, uncoated bubbles remain spherical and the interface undergoes pure dilation. A bubble in ultrasound can be thought of as a forced harmonic oscillator, with a mass associated to the inertia of the liquid, and a restoring force associated to the compressed gas.^30^ The amplitude of radial oscillations increases with the forcing amplitude, *p*_a_, and is a maximum for a resonance frequency, *ω*_0_, that is inversely proportional to the size of the bubble,^31^ 
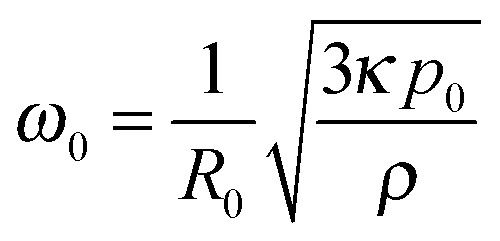
, where *R*_0_ is the resting radius, *κ* the polytropic exponent, *p*_0_ the ambient pressure, *ρ* the density of the liquid, and surface tension effects have been neglected.

The effect of surface coatings on bubble dynamics in ultrasound has been studied primarily for the case of lipid-coated bubbles.^32–35^ With a surface coating present, the surface tension of the interface changes as the surface concentration of the adsorbed species oscillates during compression–expansion. The resonance frequency of a coated bubble depends both on the surface tension and on the elasticity of the surface coating, that is, the rate of change of surface tension with changing area.^32^ For particle-coated bubbles, it is possible to relate the effective surface tension, *γ* = *γ*_0_ – *Π*, where *γ*_0_ is the surface tension of the bare interface, and *Π* the surface pressure of the particle monolayer, to the surface coverage by particles, 
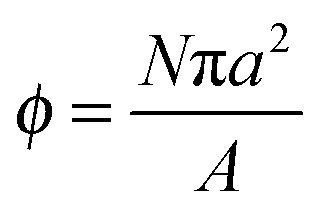
, provided that the particles can be visualised by optical microscopy.^26^ Here *N* is the number of particles in the monolayer, *a* the particle radius, and *A* the total surface area of the monolayer. The surface pressure *Π*(*φ*) can be measured for instance using a Langmuir trough.^36^

### 2.2 Shape oscillations

If an initially small perturbation of the spherical shape grows in amplitude during periodic compression–expansion through a parametric instability, the bubble undergoes shape oscillations.^29^ Shape oscillations cause non-uniform dilation of the interface, as well as shear and bending.^37,38^ Parametric instability occurs for a driving frequency *ω* = 2*ω*_*n*_, with *ω*_*n*_ the resonance frequency of a spherical harmonic distortion of order *n* (*n* > 1), given by:^40^
1
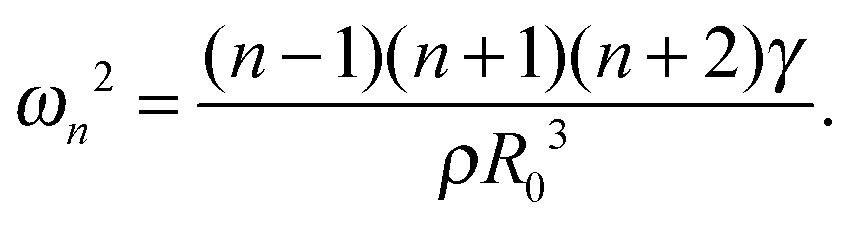
 The threshold in acoustic forcing for the onset of shape oscillations depends on the acoustic frequency, and is a minimum when the bubble is driven close to the resonance frequency for volumetric oscillations,^29^ *ω*_0_. As a result of the condition for parametric instability, shape oscillations forced at a frequency *ω* exhibit subharmonic behaviour, that is, they exhibit periodic behaviour with frequency *ω*_*n*_ = *ω*/2. Experiments on uncoated bubbles show mode selectivity depending on the bubble radius, *R*_0_, consistent with eqn (1), and subharmonic behaviour. Shape oscillations of lipid-coated bubbles have been found to also exhibit subharmonic behaviour, but in contrast to uncoated bubbles, mode selectivity was not observed.^34^ Modifications of eqn (1) have been recently proposed, to take into account the effects of shear and bending elasticity^37^ and shear viscosity.^38^

## 3 Materials and methods

### 3.1 Particle-coated bubbles

Particle-coated bubbles were made using charge-stabilised latex particles (ThermoFisher Scientific, Molecular Probes™) of 500 nm diameter. The particles were used as received. To promote adsorption to the water–air interface, the particles were suspended in an aqueous solution of 500 mM NaCl (VWR Chemicals, AnalaR NORMAPUR, 99.5%). Bubbles were made by mechanical agitation of a 0.4% w/v suspension using a vortex mixer. Ultrapure water with resistivity 18.2 MΩ cm (Milli-Q system, Millipore) was used to prepare all solutions.

### 3.2 Experimental setup

An observation chamber for optical microscopy was made of a glass slide and a glass coverslip separated by a 2 mm PDMS spacer. All the components of the observation chamber were cleaned using ethanol and rinsed using ultra pure water prior to each experiment. The chamber was placed on an inverted microscope (IX71, Olympus) equipped with 10× and 20× objectives. Ultrasound waves were excited in the observation chamber by a single-element piezoelectric transducer with resonance frequency 45 ± 3 kHz (SMD50T21F45R, Steminc) glued to the glass slide. The driving signal was generated by a waveform generator (33220A, Agilent) and amplified by a linear, radio-frequency power amplifier (AG1021, T&C Power Conversion Inc.). The dynamics of deformation were recorded at 300 000 frames per second using a high speed camera (Fastcam SA5, Photron). The image resolution at 10× and 20× magnification is 2 μm and 1 μm, respectively. The bubbles were driven for 20 or 40 cycles at a frequency of 40 kHz and a pressure in the range 100–500 kPa, as measured with a hydrophone (RP Acoustics, PVDF RP 33 s). Bubbles with radii ranging from 40 μm to 100 μm can be driven into shape oscillation with these parameters. Since the wavelength of ultrasound at 40 kHz in water is *λ* ≈ 3.75 cm, the pressure can be considered to be uniform over distances of the order of the bubble size.

### 3.3 Image analysis

We assume that the bubble shape has an axis of symmetry, so that each surface mode is characterised by a single integer *n*, corresponding to the number of undulations along the bubble contour. We exclude any experiments in which the shape can be seen to deviate from axial symmetry. To characterise the shape oscillations, the bubble's contour is tracked using image analysis routines in Matlab (MathWorks, Natick, MA, USA). A black and white threshold is applied to the image using the function *im2bw* with a threshold value defined by the function *graythresh*. The edge of the bubble is then tracked using the function *bwboundary*, and the boundary-pixels locations are saved. The centroid of the bubble is also extracted to calculate the distance between the boundary and the centroid. A polar coordinate system (*r*,*θ*) is defined, with origin at the center of mass of the bubble. The radial amplitude is obtained from contour tracking for each frame, and cast in polar coordinates as *R* = *R*(*θ*,*t*). The radial excursion relative to the resting radius *R*_0_ is defined as Δ*R*(*θ*,*t*) = *R*(*θ*,*t*) – *R*_0_. The mean bubble radius during shape oscillations is defined as2
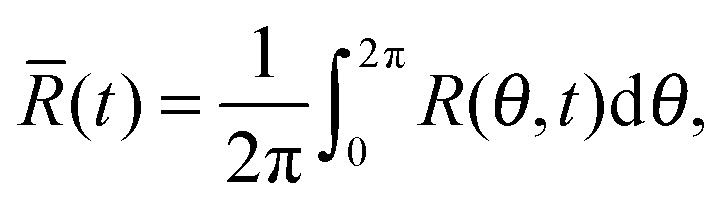
which is a measure of the volumetric oscillations of the bubble. The radial deviation from the mean radius, δ*R*(*θ*,*t*) = *R*(*θ*,*t*) – *R*(*t*), measures the deviation from spherical shape. The raw data are smoothed using Matlab's *smooth* function set to *rloess* method with a span of 5% in order to remove artefacts due to pixelisation. The pixel size limits the resolution to modes with *n* < 20. The deviation from spherical shape, δ*R*(*θ*,*t*), is then decomposed into spatial Fourier modes:3
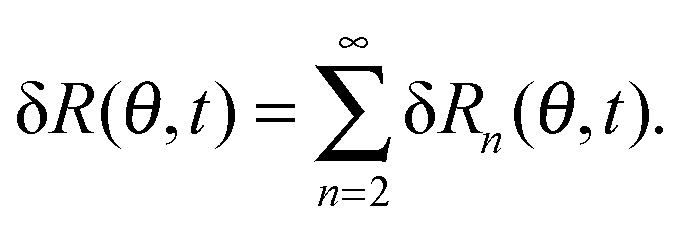
 The summation starts from *n* = 2 because *n* = 0, 1 do not represent deviations from spherical shape (*n* = 0 is the spherical mode, and *n* = 1 represents a translation of the centre of mass). The contribution from each mode, δ*R*_*n*_(*θ*,*t*), is characterised by an amplitude *A*_*n*_(*t*) and a phase *α*_*n*_(*t*):

4 δ*R*_*n*_(*θ*,*t*) = *A*_*n*_(*t*) cos[*n*(*θ* + *α*_*n*_(*t*))].

The decomposition of δ*R*(*θ*,*t*) into spatial Fourier modes fully characterises the deviation from spherical shape and the temporal evolution of the bubble shape.

## 4 Results and discussion

### 4.1 Shape oscillations of particle-coated bubbles and directional particle expulsion

**Fig. 1:**
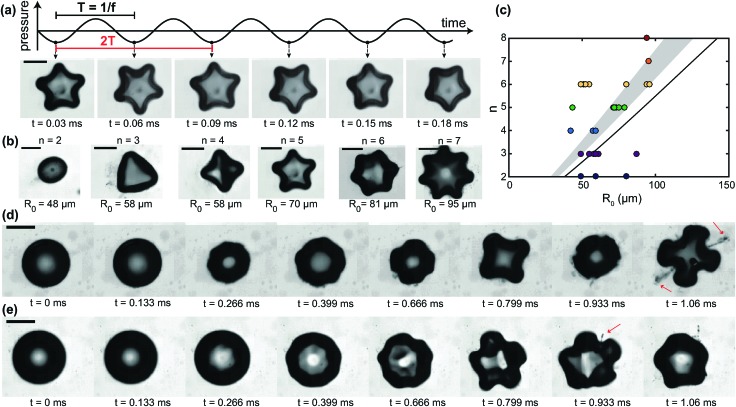
Shape oscillations of particle-coated bubbles and directional particle expulsion. (a) A particle-coated bubble undergoing shape oscillations with mode *n* = 5. The period of the ultrasonic driving is *T* = 1/*f*; the period of the shape oscillation is 2*T*, corresponding to a frequency *f*/2. (b) Examples of shape oscillations of particle-coated bubbles with different modes *n* observed in experiment. *R*_0_ is the resting radius of the bubble. (c) Mode number *n versus* resting radius *R*_0_. The experimental data (circles) show that the system does not exhibit mode selectivity. The solid line is the theoretical prediction from eqn (1) for an uncoated bubble. The shaded area is the prediction from eqn (1) for the typical range of surface tension of particle-coated bubbles. (d) Growth of shape oscillations (*n* = 4) during driving by ultrasound, followed by directional particle desorption from 2 of the 8 antinodes. (e) Another example of directional particle desorption driven by shape oscillations with *n* = 5 (see ESI,† Movie S1). All scale bars: 80 μm.

Fig. 1a shows an example of shape oscillations of a particle-coated bubble. The bubble is excited at a frequency *f* = 40 kHz. The dominant mode is *n* = 5. The acoustic pressure oscillates in time with period *T* = 1/*f*. As can be seen from the image sequence in Fig. 1a, the period of the shape oscillations is 2*T*. The observed subharmonic behaviour (with frequency *f*/2) is characteristic of shape oscillations both for uncoated^29^ and lipid-coated bubbles.^34^ Fig. 1b shows different modes of shape oscillations, with *n* = 2 to *n* = 7, that are observed in experiment for different bubbles driven at a frequency *f* = 40 kHz. In Fig. 1c we report the observed mode number *n* as a function of the initial bubble radius *R*_0_ (circles) at a fixed frequency *f* = 40 kHz. In the range of bubble sizes used in our experiments, *R*_0_ ≈ 40–100 μm, surface modes with *n* = 2 to *n* = 8 are observed, with no apparent dependence on the bubble size. The predicted dependence of mode number on resting radius for an uncoated bubble, computed from eqn (1) with *γ* = 72 mN m^–1^, is shown for reference (solid line). The shaded area corresponds to the typical range of values of surface tension for a particle–laden interface,^26^ *γ* ≈ 30–50 mN m^–1^. The large scatter in the experimental data is not captured by the models including the rheological properties of the monolayer.^37,38^ The effect of the vicinity of the wall is to decrease the resonance frequency relative to eqn (1)^39^ but this also does not explain the scatter in the experimental data. Variability in properties of the coating, for instance differences in the initial surface coverage, is a possible reason why mode selectivity is suppressed, as observed for lipid-coated bubbles.^34^ Other possible mechanisms include plastic behaviour of the coating or non-continuum effects, due to the extremely large strain rates applied (10^4^ s^–1^).

Shape oscillations direct particle expulsion from the antinodes, as we reported previously.^26^ While the observed pattern of particle expulsion typically follows the symmetry of the non-spherical mode, in general it does not have an exact 2*n*-fold symmetry. In many cases, desorption preferentially occurs only from some of the antinodes, as shown in Fig. 1d and e. In Fig. 1d, the bubble undergoes shape oscillations with *n* = 4, and plumes of particles are expelled predominantly from two of the antinodes, indicated by the arrows. In Fig. 1e, shape oscillations with *n* = 5 promote the expulsion of a single plume of particles (indicated by the arrow) from one of the antinodes (see ESI,† Movie S1). The antinodes are the locations where the radial excursion, Δ*R*, and therefore the radial velocity, ** ∼ Δ*Rω*, and the radial acceleration of the interface, *R* ∼ Δ*Rω* ^2^, are a maximum. The rate of change of area is also a maximum at the antinodes. The mechanisms that govern particle desorption from the antinodes will be discussed in Section 4.4. We first analyse the selectivity of desorption from certain antinodes by performing a mode decomposition of the bubble shape.

### 4.2 Mode decomposition of shape oscillations

**Fig. 2:**
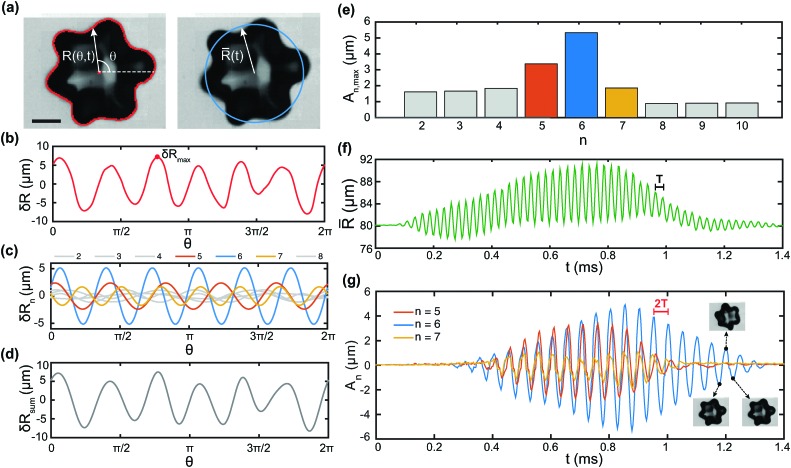
Mode decomposition of shape oscillations. (a) Image analysis gives the bubble contour and centre of mass, from which the radial amplitude *R*(*θ*,*t*) is obtained (left). The mean radius *R*(*t*) is computed from eqn (2) (right). Scale bar: 80 μm. (b) Deviation from spherical shape, δ*R*(*θ*) = *R*(*θ*) – *R*, for the frame shown. (c) Fourier decomposition of δ*R*(*θ*), where δ*R*_*n*_(*θ*) is the contribution of mode *n*, for the first 8 modes. (d) Deviation from spherical shape reconstructed from the sum of the first 8 modes only. (e) Maximum amplitude of the first 10 modes, showing that the three dominant modes are *n* = 5, 6, 7. (f) Time evolution of the mean radius *R*(*t*). The mean radius oscillates with period *T* = 1/*f*. (g) Time evolution of the amplitude of modes *n* = 5, 6, 7. Every second peak corresponds to the same bubble shape, consistent with subharmonic behaviour with period 2*T* (frequency *f*/2).

Fig. 2a shows a bubble undergoing shape oscillations with a dominant *n* = 6 mode. The bubble's contour obtained from image analysis, overlaid on the image in Fig. 2a, gives the radial amplitude as a function of the angular coordinate, *R*(*θ*). The mean radius for the same representative frame, *R*, is also shown. The corresponding radial deviation from spherical shape, δ*R*(*θ*) = *R*(*θ*) – *R*, is plotted in Fig. 2b. The Fourier transform of δ*R*(*θ*) reveals the contribution of different spatial modes. The amplitudes of the first 8 modes, δ*R*_*n*_(*θ*) with *n* = 2–8, are shown in Fig. 2c. While *n* = 6 is clearly the dominant mode, the amplitudes of other modes are non-negligible, particularly *n* = 5 and 7. Fig. 2d shows the reconstructed radial deviation, δ*R*_sum_, obtained by taking the sum of the first 8 modes. The reconstructed signal satisfactorily reproduces the experimental data, indicating that modes of higher order can be safely neglected. The Fourier analysis is performed on the entire image sequence to obtain the time-dependent amplitude of each mode, δ*R*_*n*_(*θ*,*t*). The maximum amplitude in time for each mode, *A*_*n*,max_ = max[*A*_*n*_(*t*)], with *A*_*n*_(*t*) defined in eqn (4), is shown in Fig. 2e. We focus on the three modes with the largest amplitudes, *n* = 5, 6, and 7, for the analysis of the time-dependent behaviour. Fig. 2f shows the time evolution of the mean radius, *R*(*t*). The mean radius oscillates in time as the bubble undergoes volumetric oscillations driven by the ultrasound wave. The oscillations are at the frequency of the acoustic driving, *f* = 40 kHz, which corresponds to a period *T* = 1/*f* = 25 μs. The observation that the oscillations are not around a constant value of the mean radius is likely due to an experimental artefact: since the bubble is not surrounded by an unbounded fluid, but is in contact with the solid wall of the sample cell, during oscillations it flattens against the wall. As a consequence, the projection of the shape in the observation plane is no longer representative of the bubble volume. Fig. 2g shows the time evolution of the mode amplitude, *A*_*n*_, for *n* = 5, 6, and 7. The three modes develop at *t* ≈ 0.3 ms. All the modes exhibit subharmonic behaviour, as they oscillate with a period 2*T*, as expected.

### 4.3 Temporal evolution of non-spherical modes

**Fig. 3:**
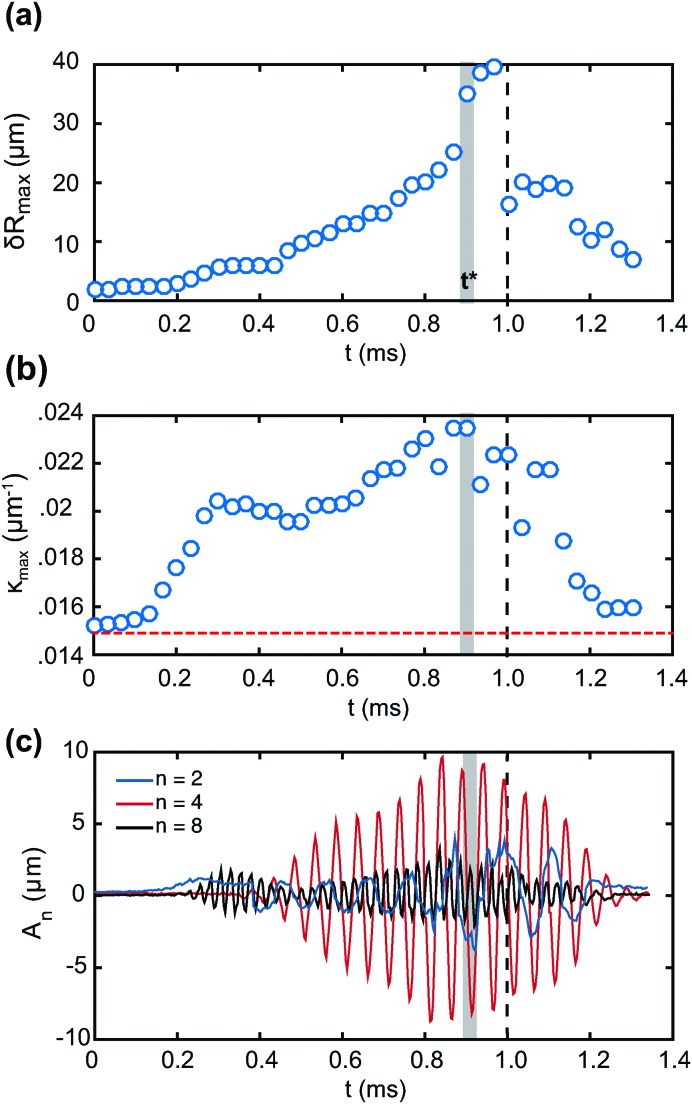
(a) Time evolution of the maximum deviation from spherical shape. The shaded area corresponds to the desorption event and the dashed line corresponds to the end of the ultrasound driving. (b) Time evolution of the maximum interface curvature. The horizontal dashed line represents the curvature of the bubble at rest. (c) Time evolution of the amplitude of the three main modes, *n* = 2, 4 and 8. The three modes exhibit different frequencies, indicating that the dynamics are non-linear.

We perform a mode decomposition for the experiment shown in Fig. 1d, to reveal the role of the interplay of different modes in determining the pattern of particle expulsion. Fig. 3a shows the temporal evolution of the maximum deviation from spherical shape, δ*R*_max_(*t*), defined as the maximum with respect to *θ* of δ*R*(*θ*,*t*) (see Fig. 2b). The maximum deviation from spherical shape increases during ultrasonic driving, until it reaches a maximum in time. It then decays after the driving stops at *t* = 1 ms. The time at which desorption occurs, t*, is represented by the shaded area. Desorption occurs just before δ*R*_max_ reaches its maximum in time. From the bubble contour, *R*(*θ*,*t*), we compute the local interface curvature *κ*(*θ*,*t*), defined as 
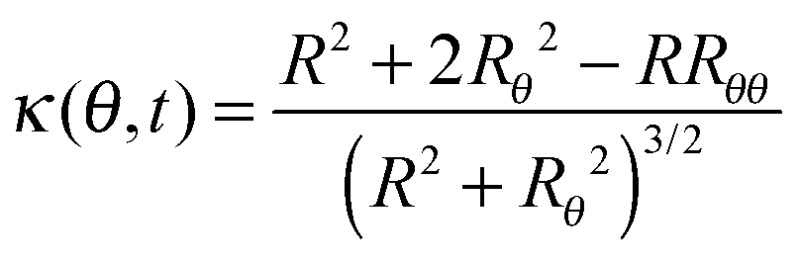
, where the subscript *θ* denotes the partial derivative with respect to *θ*. We take the maximum with respect to *θ* to obtain *κ*_max_(*t*). Fig. 3b shows the evolution of *κ*_max_ as a function of time. The horizontal dashed line shows the initial curvature of the interface, corresponding to the resting radius *R*_0_. Desorption, marked by the shaded area at time t*, occurs when the interface curvature is also a maximum. To explain the occurrence of desorption only from certain antinodes, we now analyse the contributions of different modes. Fourier analysis reveals that, in addition to the clearly visible mode *n* = 4 (see Fig. 1d), modes *n* = 2 and *n* = 8 also have significant amplitude. Fig. 3c shows the temporal evolution of the mode amplitude *A*_*n*_ for *n* = 2, 4 and 8. Mode *n* = 8 develops first, with a frequency *ω*_8_ = *ω*_0_. Modes *n* = 2 and *n* = 4 develop from *t* ≈ 0.4 ms, with frequencies *ω*_4_ = *ω*_0_/2 and *ω*_2_ = *ω*_0_/4. These behaviours indicate that the regime of shape oscillations is in this case non-linear, in keeping with the fact that the amplitude of deviation from spherical shape is larger than for the experiment of Fig. 2g. Sub-harmonic and harmonic mode coupling have been reported for acoustically driven bubbles in the non-linear regime, with resonant energy transfer typically from higher- to lower-order modes.^41^ The amplitude of all three modes is a maximum at, or near, the time when desorption occurs, *t* = t*. The presence of different modes partly explains why the desorption pattern does not simply follow the symmetry of the dominant mode, *n* = 4.

**Fig. 4:**
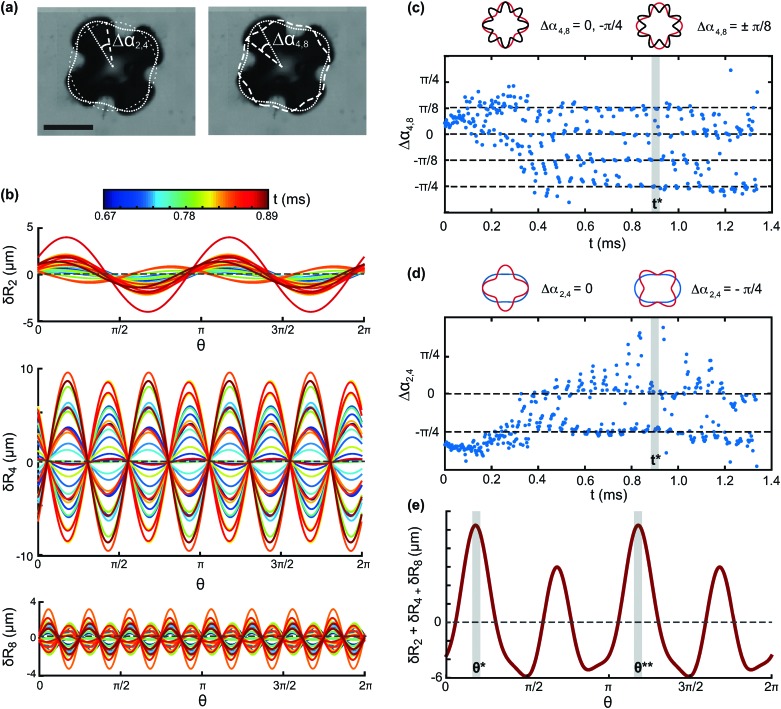
(a) Contours of modes *n* = 2, 4 and 8 overlaid on the overall bubble shape. Δ*α*_2,4_ and Δ*α*_4,8_ are the phase differences between the modes. Scale bar: 80 μm. (b) Time evolution of the three modes. Different times correspond to different colour (see colorbar). (c) Time evolution of the phase difference between modes 4 and 8, Δ*α*_4,8_. The two modes align with their antinodes in phase. The shaded area corresponds to the time of desorption. (d) Time evolution of the phase difference between modes 2 and 4, Δ*α*_2,4_. The phase difference behaves erratically in time, but the two modes are aligned at the time of desorption (shaded area). (e) Superposition of modes *n* = 2, 4 and 8 at the time of desorption. The shaded areas mark the locations of the two desorption plumes, which are found to correspond to the locations of maximum deviation from spherical shape.

To understand how the interplay of the three modes leads to the desorption pattern shown in Fig. 1d, we examine their spatial alignment. Fig. 4a shows the contours of the three modes *n* = 2, 4 and 8 at time *t* = 0.83 ms, overlaid on the image of the overall bubble shape. The phase differences Δ*α*_2,4_ and Δ*α*_4,8_ are also shown, where we have defined the time-dependent phase difference between modes *p* and *q* as Δ*α*_*p*,*q*_(*t*) = *α*_*p*_(*t*) – *α*_*q*_(*t*). In Fig. 4b we plot each of the modes δ*R*_*n*_(*θ*), with *n* = 2, 4 and 8, for different times. The amplitude of mode 2 increases monotonically in time, and the phase of the mode changes, that is, the mode drifts along the interface. For modes 4 and 8, the amplitude changes non-monotonically in time, and the phase does not change significantly. Because the modes under consideration are second harmonics of each other (*p* = *n*, *q* = 2*n*) a condition for the phase difference that gives alignment of the antinodes (mod 2π) can be obtained:5
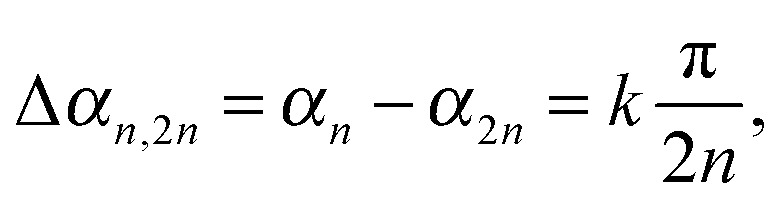
with *k* = 0, ±1, ±2, …,2*n*. Of the 4*n* possible configurations that give alignment of antinodes, 2*n* correspond to alignment of maxima, and 2*n* to alignment of minima.

The temporal evolution of Δ*α*_2,4_ and Δ*α*_4,8_ is shown in Fig. 4c and d. The two phase differences change rapidly during the initial 0.4 ms, as modes 2 and 4 are still developing. After *t* ∼ 0.4 ms, modes 2, 4 and 8 are all present. For modes 4 and 8, the phase difference Δ*α*_4,8_ takes the values 0, 
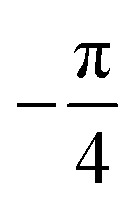
, corresponding to alignment of maxima, and 
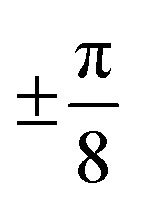
, corresponding to alignment of minima, as shown in the schematic in Fig. 4c. The modes are aligned with their antinodes in phase. For modes 2 and 4, the phase difference Δ*α*_2,4_ exhibits a more erratic behaviour. Alignment is observed for 
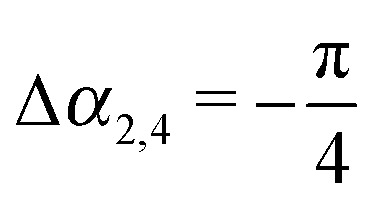
, corresponding to alignment of minima, whereas there are large fluctuations around Δ*α*_2,4_ = 0 corresponding to alignment of maxima (see the two configurations in the schematic in Fig. 4d). At the time where desorption occurs, t*, marked by the shaded area, modes 2 and 4 temporarily align also at Δ*α*_2,4_ = 0. At this time, where the antinodes of all three modes are aligned, the sum δ*R*_2_ + δ*R*_4_ + δ*R*_8_ shows two maxima at the angular locations, θ* and θ**, where the two main desorption plumes are observed. Mode 4 contributes the most to the deviation from spherical shape, and reaches an amplitude of almost 10 μm at desorption (see Fig. 3c). Mode 2, whose amplitude is 4 μm at *t* = t*, imposes the selectivity on two of the antinodes.

### 4.4 Mechanisms of directional particle desorption

**Fig. 5:**
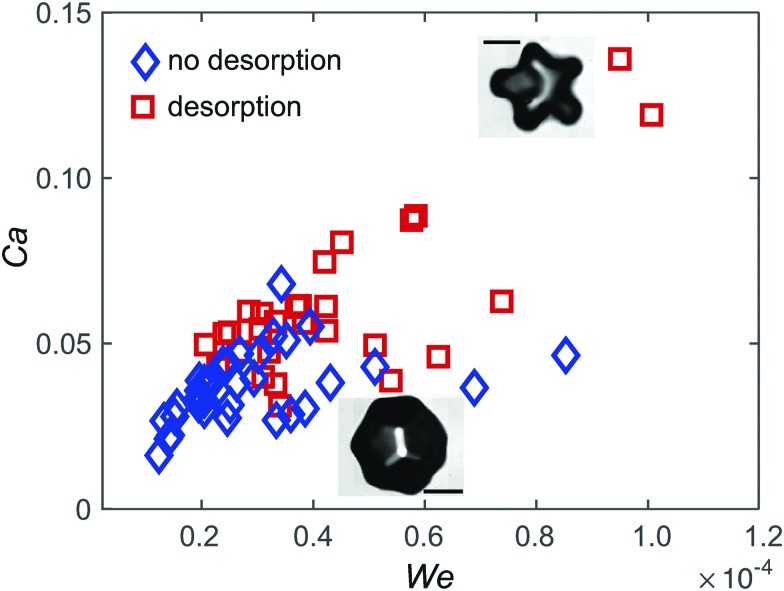
Phase diagram for directed particle desorption driven by shape oscillations. The Weber number, We, and the capillary number, Ca, compare respectively the inertial and viscous force on a particle with the capillary force holding the particle at the interface. The diamonds correspond to experiments where desorption is not observed, the squares to desorption events. The scale bars in the insets are 80 μm.

The forces that can contribute to promoting desorption in this system are the inertia of the particles, viscous drag on the particles, and the contact forces between neighbouring particles. When the component normal to the interface of the net force on a particle exceeds the capillary force holding the particle at the interface, the particle is expelled. As the three forces reach their maximum value during different stages of the shape oscillation, we now examine their temporal evolution and relative magnitude. We can write the local amplitude of the radial excursion at an antinode, Δ*R*_a_(*t*), as Δ*R*_a_(*t*) ∼ Δ*R*_max_ sin(*ωt*). Recall that Δ*R*(*θ*,*t*) = [*R*(*t*) – *R*_0_] + δ*R*(*θ*,*t*). The interface velocity at the antinode is then **_a_ ∼ Δ*R*_max_ *ω* cos(*ωt*), and the acceleration *R*_a_ ∼ –Δ*R*_max_ *ω* ^2^ sin(*ωt*). The viscous drag on a particle at an antinode, *F*_d_ ∼ –*ηa*_a_, is directed outwards when the interface is retracting, and its magnitude is a maximum when the interface is going through the mean radius *R*. The net inertial force on a particle, *F*_i_ ∼ Δ*ρa* ^3^ *R*_a_, is in anti-phase with the radial excursion amplitude. This force is directed outwards and has maximum magnitude when the radial excursion is a minimum. Due to the 
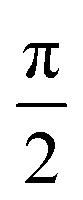
 phase difference between **_a_ and *R*_a_, the inertia of a particle is zero when the viscous drag is a maximum, and *vice versa*. The magnitudes of the two forces can be compared with the capillary force, *F*_c_ ∼ *γ*_0_ *a*, resulting in two non-dimensional numbers: the Weber number, We, based on the acceleration of the interface, and the capillary number, Ca, based on the viscous drag force on a particle:6
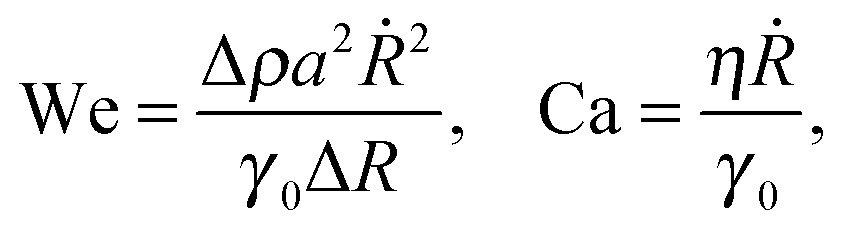
where we have used the fact that *F*_i_ ∼ Δ*ρa* ^3^Δ*R*_max_ *ω* ^2^ = Δ*ρa* ^3^ **_a_^2^/Δ*R*_max_. Fig. 5 reports the phase diagram for particle desorption in the (We, Ca) plane, with We and Ca calculated from the maximum values of Δ*R*_max_ and **_a_ for each experiment. The observed qualitative trend is that there is a minimum threshold in Ca and We for desorption to occur, but the transition is not sharp, possibly due to the variability in initial surface coverage. Furthermore, the values of Ca and We are both much smaller than 1 even when particle desorption is observed. Particle desorption under the effect of gravity has been previously reported to occur despite the Bond number, which compares gravitational forces with surface tension forces, being low.^24^ This phenomenon was ascribed to the collective effect due to the surrounding particles in the monolayer, and is consistent with desorption from points of high curvature, as observed also in our experiments.^26^ While the inertia of one particle is not sufficient to promote desorption, the sum of the inertial forces on all the particles in the monolayer acting on the particle at the point of maximum curvature causes desorption. In other words, for desorption to occur, the number of particles at the antinode, *N* should be such that *N*We > 1. From Fig. 5 we see that the number of particles participating in the collective effect should be *N* ∼ 10^4^–10^5^. For 500 nm particles on an antinode with radius of curvature 40 μm (corresponding to the maximum curvature in Fig. 3b), at a surface coverage *φ* ≈ 0.5, we get *N* ≈ 25 × 10^4^, consistent with the requirement for desorption. For the viscous drag, it is expected that hydrodynamic interactions between particles would result in an effectively higher drag force, but we cannot provide a simple estimate of collective effects in this case. We also expect an enhanced viscous dissipation due to the high-frequency oscillatory motion of the particles, which causes unsteady viscous effects.^42^ The correction to the quasi-steady drag scales as 
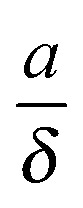
, where 
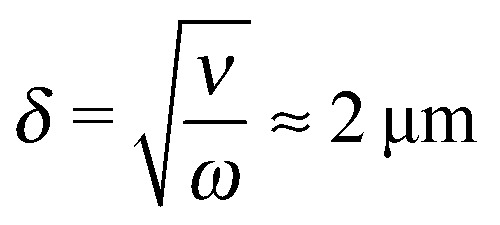
 is the diffusive length, with *ν* = 10^–6^ m^2^ s^–1^ the kinematic viscosity of water. For particles with radius *a* = 0.5–2 μm the correction ranges from a factor of 1.1 to a factor of 2 of the quasi-steady drag. On the other hand, since the particle is only partially immersed in the liquid, the quasi-steady drag could be decreased by a factor of the same order of this correction. We therefore did not attempt to evaluate the viscous forces more accurately. From visual estimation of the timing of particle expulsion relative to the expansion/retraction dynamics of the antinode, we tentatively exclude viscous drag as a dominant mechanism, because particles do not seem to be expelled at the time when the viscous drag is expected to be a maximum.

**Fig. 6:**
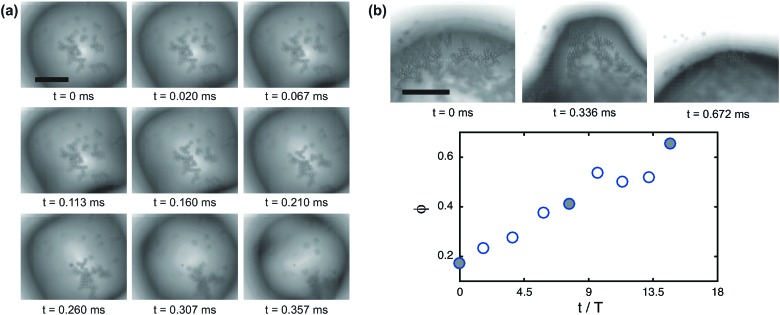
Migration of particles to the antinodes of shape oscillations. (a) Image sequence of a bubble undergoing shape oscillations (*n* = 4). Over 20 cycles of oscillations the particles accumulate at one of the antinodes (see ESI,† Movie S2). Scale bar: 40 μm. (b) Particle tracking shows the net increase in surface coverage *φ* at an antinode (*n* = 5) over several periods of oscillation. The time axis is normalised by the period of the ultrasound driving, *T*. The frames in the image sequence correspond to the filled symbols in the graph. Scale bar: 50 μm.

The third force that contributes to particle expulsion is due to the tangential stress, or surface pressure, *Π*. The tangential stress results in a non-zero normal component of the force on a particle when the interface is curved. The normal component increases with increasing curvature of the interface,^5^ consistent with the observation that desorption occurs when the curvature is a maximum (see Fig. 3b). The surface pressure *Π* depends on the surface coverage by particles, which is expected to be non-uniform over the surface of a bubble undergoing shape oscillations, since the rate of change of area is non-uniform. We performed high-magnification visualisations of the particle distribution at an antinode using larger particles (4 μm or 5 μm diameter). The bubble shown in Fig. 6a has a resting radius *R*_0_ ≈ 60 μm and exhibits shape oscillations with a dominant mode *n* = 4 at *f* = 43 kHz. Over 20 cycles of shape oscillations, the particles initially located at the centre are seen to migrate to one of the antinodes (see ESI,† Movie S2). In Fig. 6b we report the surface coverage *φ* at an antinode as a function of the number of periods of oscillations for a bubble with radius *R*_0_ ≈ 110 μm undergoing shape oscillations with mode *n* = 5 at *f* = 23 kHz. The frames in the image sequence correspond to the solid symbols in the graph. The local surface coverage at the antinode increases from *φ* ≈ 0.2 to *φ* ≈ 0.6 due to particle migration. The migration of particles to the antinodes further contributes to localising desorption, since the surface pressure *Π* is larger due to the local increase in surface coverage, and its effect on desorption is amplified by the larger curvature at these locations. The mechanism causing migration of particles to the antinodes remains unclear at this stage, and is the subject of current investigation. A possible mechanism is capillary interactions: the particles generate a deformation of the interface due to roughness of the contact line,^43^ which interacts with the curvature gradient at the antinodes.^44^

## 5 Conclusions

We have studied the desorption of colloids from the interface of particle-coated bubbles undergoing shape oscillations during ultrasonic driving. We investigated whether the shape oscillations of particle-coated bubbles exhibit mode selectivity, that is, if different mode numbers are observed for bubbles of different sizes. Selectivity is not observed, in contrast with uncoated bubbles, but in agreement with observations for lipid-coated bubbles. Desorption of colloids from the interface is directional and localised at the antinodes of the shape oscillations. The antinodes are the locations where the radial acceleration of the interface, the interface curvature, and the rate of change of area have their maximum value. Desorption typically occurs only from a subset of the 2*n* antinodes of a bubble undergoing shape oscillations with mode *n*. Decomposition of the bubble shape into spatial Fourier modes reveals the occurrence of different modes in addition to the dominant mode that is clearly visible with the naked eye. The interplay of modes of different order results in preferential desorption from the antinodes where the modes are in phase. Several mechanisms are likely to promote particle desorption from the antinodes. Firstly, the inertia of the particles can drive desorption through a collective effect at the points of high curvature, the so-called “keystone” effect.^24^ Secondly, the surface pressure is a maximum at the antinodes, where the rate of change of area is a maximum. In this case, the particles are pushed out of the interface because of the excluded volume constraint (the particles cannot overlap). In addition, our experiments revealed migration of particles to the antinodes during shape oscillations. As a result, the accumulation of particles at the antinodes further enhances the contribution of the surface pressure in driving desorption. Our results on controlled desorption of colloidal particles from ultrasound-driven bubbles may find applications in drug delivery, catalysis, and sonochemistry.
